# Impact of dietary phosphorous in diploid and triploid Atlantic salmon (*Salmo salar* L.) with reference to early skeletal development in freshwater

**DOI:** 10.1016/j.aquaculture.2018.02.049

**Published:** 2018-03-01

**Authors:** M.A. Smedley, H. Migaud, E.L. McStay, M. Clarkson, P. Bozzolla, P. Campbell, J.F. Taylor

**Affiliations:** a Institute of Aquaculture, University of Stirling, Stirling, Scotland, UK; b BioMar, Grangemouth, Scotland, UK

**Keywords:** Triploid, Bone mineralisation, Nutrition, Minerals, Vertebral deformity

## Abstract

In order to assess the effect of dietary phosphorus (P) in reducing vertebral malformations and improving freshwater (FW) performance in triploid Atlantic salmon (*Salmo salar*), both triploid and diploid Atlantic salmon were fed three different dietary P inclusion levels (low: 4.9, medium: 7.7, and high: 9.7 g available P kg^−1^) from first feeding until smolt. Somatic and skeletal response was assessed at fry (~0.5 g), parr (~5 g) and smolt (~45 g) stages. Triploid parr initially grew faster on the high P diet, while groups fed low P resulted in a significantly higher weight at smolt. Image analysis of double stained Alcian blue and Alizarin red S fry revealed that low P fed triploid fish presented less well mineralised vertebrae, and significantly more malformed vertebrae in both parr and smolt stages following x-ray radiographic assessment. Triploid parr fed high and medium P had similar numbers of malformed vertebrae relative to their diploid counterparts but greater numbers than at smolt. Low P fed triploids had the highest prevalence of jaw and vertebral malformations as well as the highest number of deformed vertebrae in the central caudal vertebral region, which was more pronounced at parr than at smolt. Shorter vertebrae dorso-ventral lengths were observed throughout the spinal column (R1–R4) in parr fed low P and only in the caudal region (R3) at smolt. In parr, both ploidies showed reduced phosphate homeostasis protein *fgf23 gene* expression in vertebrae when fed low P diets, while triploids showed greater down-regulation of osteogenic factors (*alp*, *opn* and *igf1r*) between diets relative to diploids, suggesting possible greater active suppression of mineralisation and reduced osteogenic potential in triploids. No effects of diet or ploidy on gene expression were evident at smolt. Comparisons between development stages suggest early P supplementation in triploids is crucial for skeletal development. Ultimately, reducing vertebral deformities observed at smolt with higher P supplementation in triploids could contribute towards improving skeletal performance and welfare of the stocks in the marine phase.

## Highlights

• Feeding higher dietary P throughout freshwater significantly reduced occurrence of vertebral malformations in triploid Atlantic salmon.
• Triploid growth was improved with higher dietary P within the fry to parr stages but reduced growth from parr to smolt.
• Higher dietary P supplementation impeded diploid growth throughout freshwater.

## 1 Introduction

Recent years have seen increased commercial interest in the use of artificially induced triploid Atlantic salmon (*Salmo salar*). Artificial triploid induction, assuming use of all female stocks, in Atlantic salmon has been proposed to eliminate adverse impacts of farmed escapees breeding with wild populations (Glover et al., 2013), improve potential for greater harvest weights (Fjelldal et al., 2015; Smedley et al., 2016) and have been suggested to widen windows of seawater transfer (Taylor et al., 2012). Awareness of suboptimal culture conditions relative to diploids including mixed ploidy rearing (Taylor et al., 2014) and high environmental temperature (Atkins and Benfey, 2008) combined with low oxygen levels (Hansen et al., 2015; Sambraus et al., 2017) have improved production prospects. However, recurrence of vertebral and jaw malformations have hindered wider commercial adoption due to concerns over welfare (Fraser et al., 2012a). Aetiology of skeletal deformities in farmed triploids are largely associated with higher suboptimal egg incubation temperature (Fraser et al., 2014c), accelerated growth (Leclercq et al., 2011; Taylor et al., 2012; Taylor et al., 2014) in association with dietary deficiencies (Fjelldal et al., 2015). In particular, high prevalence of skeletal deformities that are typically observed in triploid Atlantic salmon at harvest, have been shown to be associated with vertebral deformities already present at sea transfer (Fjelldal and Hansen, 2010; Fjelldal et al., 2015; Smedley et al., 2016). Hence, environmental and particularly nutritional requirements in freshwater rearing of triploid Atlantic salmon must be addressed.

Repeated observation of lower condition factors in triploid Atlantic salmon (Fjelldal and Hansen, 2010; Taylor et al., 2011; Taylor et al., 2012) may suggest a lower total body lipid content (Herbinger and Friarrs, 1991). Alternatively this could suggest an increased deposition of skeletal components over muscle, which alongside increased skeletal malformation prevalence, illustrate the potential for higher mineral requirement in triploid bone formation. Dietary phosphorous (P) deficiency is a primary nutritional risk factor for skeletal development in fish (Lall and Lewis-McCrea, 2007) including Atlantic salmon (Asgard and Shearer, 1997; Fjelldal et al., 2012a) and is also an essential structural component of nucleic acids, ATP and cell membrane phospholipids. Alongside calcium (Ca) it is the main mineral component of bones, teeth and scales and, unlike calcium, must be met by diet in fish (Lall, 2003). The minimum recommended requirement for P in salmonids is 8 g total P kg^−1^ (NRC, 2011). Long term effects of impeded growth, poor vertebral mineralisation and increased prevalence of vertebral malformations have been observed in diploid Atlantic salmon juveniles fed levels below these recommendations (Fjelldal et al., 2009; Fjelldal et al., 2012a). Skeletal malformations observed in diploid Atlantic salmon at harvest are shown to originate from nutritional P deficiency in freshwater (Fjelldal et al., 2012a), which has also been implicated in triploid Atlantic salmon (Smedley et al., 2016). Fjelldal et al. (2015) clearly showed fewer vertebral and jaw malformations at seawater transfer and harvest in triploid Atlantic salmon fed higher P inclusion in freshwater (9.4 vs. 7.1 g total P kg^−1^). Results suggest freshwater developmental stages in triploids require higher P inclusion for correct skeletal development.

One of the factors that may influence the observed differences in skeletal development and associated nutritional requirement between triploids and diploids is growth rate. Vertebral mineralisation can be compromised during periods of accelerated growth (Hansen et al., 2010). Elevated rearing temperatures for Atlantic salmon juveniles result in faster growth accompanied by poor vertebral mineralisation through disrupted bone and cartilage formation (Ytteborg et al., 2010). Instances of faster growth alongside higher incidence of vertebral deformities in triploid Atlantic salmon have been reported (Leclercq et al., 2011; Taylor et al., 2012) and may indicate dietary requirements could be greater for triploids than their diploid counterparts. Higher P and protein diets have been shown to stabilise severity of malformations and sustain faster growth in seawater for triploid Atlantic salmon (Smedley et al., 2016). Faster growth in diploids coincide with increased bone density and expression of *igf-I receptor* in bone (Wargelius et al., 2005), where local expression may be associated with initiating extracellular matrix (ECM) production. Hence, accelerated growth factors anticipated in triploid freshwater growth may impact regulation of mineralisation of the ECM. Environmental factors that induce accelerated growth in diploid Atlantic salmon such as high temperature, have also led to vertebral fusions and associated upregulation of *matrix metalloproteinase 13* (*MMP-13*) (Wargelius et al., 2010) and downregulation of osteogenic marker *collagen typ Iα1* (*Col1a1*) (Ytteborg et al., 2010). MMP-13 is a matrix metalloproteinase which is involved in the degradation of the extracellular matrix (ECM) associated with chronic inflammatory responses characterised by bone remodelling and leading to deformity. Collectively, given the known differences in growth performance between diploids and triploids these osteogenic biomarkers may be useful in elucidating mechanisms for triploid associated skeletal deformity and the role of dietary P in homeostatic mechanisms of bone growth.

Hydroxyapatite, the key mineral component of bone, synthesis is dependent on phosphate (PO_4_^3−^) and calcium (Ca^2+^) homeostasis which is systemically regulated primarily through circulating parathyroid hormone (PTH), 1,25-dihydroxyvitamin D [1,25(OH)2D] and fibroblast growth factor 23 (FGF23; Martin et al., 2012). These factors regulate PO_4_^3−^ intestinal absorption, remodelling in bone and excretion in kidney, through sodium-phosphate cotransporter (Npt2a) activity, according to demand and availability. Increased kidney Npt2a activity is observed under periods of dietary PO_4_^3−^ restriction in rainbow trout (Sugiura et al., 2003), however, little research has been conducted on these P-bone homeostasis pathways in salmonids, let alone triploid fish (Fjelldal et al., 2015). In other vertebrates, FGF23 secretions are known to be directly induced through expression of *fgf23* from osteoblasts and osteocytes, the mineralising osteogenic cells in bone, and promote renal PO_4_^3−^ resorption with coordination of [1,25(OH)2D] (Martin et al., 2012). In addition, alkaline phosphatase (ALP) and osteopontin (OPN) are both markers for extracellular mineralisation around osteoblasts. ALP provides osteoblasts with PO_4_^3−^ by dephosphorylating exogenous β-glycerophosphate (Beck et al., 2000; Pombinho et al., 2004). In turn, the presence of free PO_4_^3−^ stimulates OPN secretions to the osteoblast ECM to promote osteogenic function (Beck et al., 2000). As such, ALP and OPN and their corresponding transcription factors, are collectively strong indicators of osteogenic activity in the presence of free PO_4_^3−^ and may also be useful markers to determine ploidy differences in response to dietary P.

The present study aims to investigate dietary P supplementation in diploid and triploid Atlantic salmon siblings with an emphasis on impacts at three freshwater life stages: fry, parr and smolt. Growth performance, skeletal malformation, mineral composition and mRNA expression of key bone homeostatic genes were analysed.

## 2 Materials and methods

### 2.1 Fish stock

**Fig. 1:**
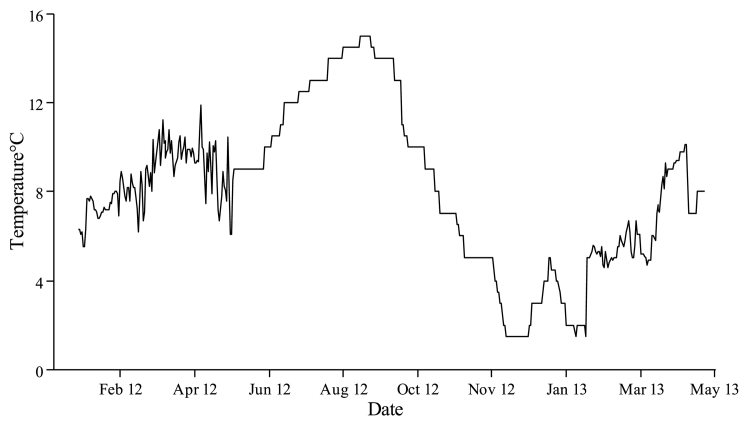
Temperature (°C) from the beginning of the trial (January 2012) until smoltification (May 2013).

On January 19, 2012, sibling groups of diploid and triploid Atlantic salmon eggs (20 dams & 5 sires, Atlantic QTL-innOva® IPN) were supplied from AquaGen (Norway) to Howietoun Fish Farm, Stirling (56°N, 4°W) at 395 degree-days post-fertilisation (^○^DPF). Triploidy was induced using a hydrostatic pressure shock of 9500 psi applied 300 °min post-fertilisation for 50 °min at 8 °C (Taylor et al., 2011). From fertilisation to point of shipping ova were incubated in upwelling silos at temp range 3.2–8.0 °C. Prior to shipping, equal numbers per family (1500/family) and ploidy were pooled. Eyed eggs (5000/tank, 250/family) were equally split between 12 × 250 L tanks (6 per ploidy) and reared under constant darkness at 8.7 ± 1.0 °C until first feeding (929 ^○^DPF; March 26, 2012). Fry were transferred at 1387 ^○^DPF (~0.43 g) to the Niall Bromage Freshwater Research Facility (NBFRF), Stirling (56°N, 4°W) where they were maintained in 12 × 980 L covered circular tanks. Fish numbers were periodically reduced by randomly netting and removing 50% of the population (average fish no. per tank: 5000 eyed ova; 2500 @ 0.43 g; 1250 @ 5 g; 625 @ 30 g; 414 at smolt) to maintain stocking density <30 kg m^−3^. Fish were reared under continuous light (LL) until August 31, 2013 followed by simulated natural photoperiod and ambient water temperature (Fig. 1) until smoltification (~45 g, 3321 ^○^DPF, April 24, 2013). Smoltification was verified using smolt index scoring (all tanks scoring mean 4.0 on 24th April) according to Sigholt et al. (1995) following 400 degree days of increasing daylength post-winter solstice. All fish were vaccinated with PHARMAQ Alphaject 2.2 on February 26, 2013. Triploid rate was verified through erythrocyte measurements. Red blood smears were prepared from blood collected from the caudal vein using heparinised syringes from fish at 5 g (n = 100/ploidy, 16–17 fish per tank (n = 6/ploidy), representing 1.3% of total population number (7500 at 5 g). After air drying, slides were fixed in 100% methanol and then placed into Giemsa stain for 10 min. Erythrocyte length and diameter were measured at 100× magnification using image capture (ImagePro Software). A total of 30 randomly chosen nuclei per slide were measured to the nearest 0.01 μm. Diploid control groups had significantly smaller nuclear lengths with no overlaps with pressure shock triploid groups (2N: 7.3–7.5 μm; 3N: 9.2–10.1 μm) confirming that all fish that were subjected to hydrostatic pressure shock were likely to be triploids.

### 2.2 Experimental setup and sampling

Duplicate groups of diploid and triploid fish were fed from first feeding until smoltification, one of three different diets using clockwork belt feeders according to manufacturer's tables in relation to biomass and water temperature. Each diet was formulated (Table 1) by BioMar UK Ltd, Grangemouth, Scotland and contained graded levels of available dietary P: 4.9 g kg^−1^ (low, LP), 7.7 g kg^−1^ (medium, MP) and 9.7 g kg^−1^ (high, HP). Differential P levels were achieved through inclusion of monosodium phosphate (MSP), while all other ingredients were modified slightly to account for increased MSP addition. It is noted certain micronutrients (V, Mn, Fe and Co) increased with MSP addition, and is likely related to the slight alteration in fish to plant protein raw material ratio to allow space for MSP addition. Growth parameters including fork length (±1 mm), weight (±0.1 g), condition factor (K) and externally visible malformations including spinal (shortening, scoliosis/lordosis) and jaw (lower jaw downward curvature, side twisted lower jaw), were assessed on a monthly basis following anaesthesia (Tricaine, 50 ppm, PHARMAQ UK) of randomly selected fish (50/tank, n = 2) until trial termination where all remaining fish/tank (385–440) were visually assessed. Whole fish samples were taken at 5 g (parr; n = 50/tank) and 45 g (smolt; n = 35/tank) following lethal anaesthesia (Tricaine, 1000 ppm) and stored at −20 °C until x-radiography and whole body mineral concentration analysis. An additional 10 fish per treatment (n = 5/tank) were euthanised through lethal anaesthesia and fixed in 10% neutral buffered formalin (NBF) at 3 and 8 weeks post-first feeding for whole mount staining. At the parr and smolt stages 12 fish per treatment (n = 6/tank) were also euthanised and vertebral sections were taken from the central region below the dorsal fin (vertebra 18–32), which is known to have higher susceptibility to deformity development (Fraser et al., 2014c; Fjelldal et al., 2015), and stored in RNA later for 24 h prior to freezing at −20 °C for gene expression analysis. All experimental procedures and husbandry practices used in the present study were conducted in compliance with the Animals Scientific Procedures Act 1986 (Home Office Code of Practice) in accordance with EU regulation (EC Directive 86/609/EEC) and approved by the Animal Ethics and Welfare Committee of the University of Stirling.

**Table 1:** Formulation and analysed composition of experimental feeds.

	LP	MP	HP
*Formulation (%)*			
Fish/Crustacean meal	51.49	53.68	53.23
Plant protein concentrates	23.83	20.35	19.73
Wheat flour	10.01	9.84	9.22
Fish oil	9.68	9.50	9.43
Rapeseed oil	2.86	2.80	2.76
Monosodium phosphate	0.36	2.01	3.81
Premix^a^	1.77	1.81	1.82

*Proximate and mineral composition analysis*			
Moisture (%)^b^	6.45	6.73	6.42
Oil (%)^b^	19.34	19.64	18.77
Protein (%)^b^	52.16	51.51	51.21
Ash (%)^b^	7.87	9.03	10.20
Total P g kg^−1^^b^	13.0	16.7	19.7
Available P g kg^−1^^c^, ^b^	4.9	7.7	9.7
Soluble P g kg^−1^^d^	10.4	13.6	15.2
Total Ca g kg^−1^^b^	11.7	11.6	11.7
Total Ca:P ratio	0.89	0.69	0.59
Total Na g kg^−1^^b^	6.0	9.2	14.2
Total Mg g kg^−1^^b^	1.8	2.0	2.2
Total K g kg^−1^^b^	8.6	8.5	7.8
Total V mg kg^−1^^b^	0.6	0.9	1.2
Total Mn mg kg^−1^^b^	30	36	44
Total Fe mg kg^−1^^b^	230	330	450
Total Co mg kg^−1^^b^	0.08	0.15	0.22
Total Cu mg kg^−1^^b^	21	20	20
Total Zn mg kg^−1^^b^	250	240	250

a BioFish Premix: combination of essential vitamins, minerals and amino acids meeting NRC (2011) recommendations for Atlantic salmon.

b University of Stirling, UK. Proximate composition analysed according to methods of AOAC (2000), with total oil modified according to methods of Folch et al. (1957) and Henderson and Tocher (1992). Elemental analysis according to method of Refstie et al. (1997).

c BioMar UK, Grangemouth, UK.

d Measured by ELISA (Megaenzyme, USA) according to method & validation of Hovde (2013).

### 2.3 Vertebral assessment

#### 2.3.1 Whole mount staining for bone and cartilage

Fry (3 and 8 weeks post-first feeding, ~0.5 g) fixed in 10% NBF were double stained according to Potthoff (1984) for cartilage (Alcian blue, 8GX: Sigma Aldrich, USA) and calcium in hydroxyapatite (alizarin red S sodium salt: Alfa Aesar, USA). Stained specimens were photographed using a light tent and table with a Nikon 300S camera (Japan) with a 60 mm and F 2.8 lens, with digital images analysed using Image J (Image J 1.46r, NIH, USA) in which calcium stain as an indicator of vertebral area was assessed according to four vertebra (v) regions (R1, v1–8; R2, v9–30; R3, v31–49; R4, v50–58/59/60) as defined in Kacem et al. (1998). Individuals were not large enough for accurate quantifiable image analysis measurements until 8 weeks post-first feeding, and were only visually assessed at 3 weeks.

#### 2.3.2 Radiological assessment

Lateral view radiographs were taken at parr (5 g, n = 50/tank) and at smolt (45 g, n = 35/tank). At 5 g, radiographs were taken using mammography x-ray (Bioptics BioVision, Daax Ltd, USA; calibration: kV: 22, Exp: 15 s, mAs: 22.52) and at smolt using a standard portable x-ray unit (Celtic SMR PX40 HF; calibration: kV: 40, mAs: 32) with an extremities plate measuring 24 × 30 cm, and subsequently digitised using an AGFA CR35-X Digitizer. Images were analysed for total vertebrae number, deformity classification (Witten et al., 2009) and length: dorso-ventral diameter ratios in DICOM format in Image J (Image J 1.46r, NIH, USA). Radiological x-rays were reclassified according to severity in terms of number of deformed vertebra affected (dV) (none–mild, 0–5 dV excluding ≥3 consecutive malformed vertebrae; moderate–severe, ≥6 dV, likely to affect welfare according to Hansen et al., 2010) prior to statistical analysis. Pathology types were grouped according to changes in intervertebral spacing, compressions, fusions, radio-dense/opaque, elongation and symmetry shifts.

#### 2.3.3 Bone mineral

Of frozen carcasses collected at parr (5 g) and smolt (45 g) for radiological assessment, five whole fish were homogenised in triplicate per tank (15/tank) for 5 min in an industrial blender before a subsample (~15 g) was oven dried at 105 °C for 24 h. Selected vertebrae (5 separate fish/tank) were also removed from the 4 functional regions (3 vertebra per region per fish) at smolt and analysed for bone mineralisation (% BM; Deschamps et al., 2008). Neural and haemal arches were removed and individual amphicoelous centra had remaining flesh removed. Vertebrae were then defatted in an agitated isohexane bath for 24 h, rinsed with distilled water, oven dried for 24 h at 105 °C, weighed (*W*_*dry*_) and incinerated in a muffle furnace at 600 °C for 16 h and reweighed (*W*_*ash*_). BM (%) was calculated as: *W*_*dry*_ / *W*_*ash*_ ∗ 100. At smolt only, the dentary bone and mandible, or lower jaw, was also processed for BM%. Resulting ash was processed for mineral content by adding 5 mL of AristAR® nitric acid (HNO_3_; VWR International, USA) to the sample and digested in a MarsXpress microwave digestion system set to 10 min heating phase to 160 °C, 29 min at 160 °C and 30 min cooling phase to room temperature. Digested samples were diluted to 2% HNO_3_ and elemental concentration analysed with a Thermo X Series II Inductively Coupled Plasma Mass Spectrometer (ICP-MS) and collision cell model. Total phosphorous was measured by the molybdenum-blue colorimetric spectrophotometry method at 690 nm acid following digestion (ISO 6491-1998).

### 2.4 Gene expression

#### 2.4.1 RNA isolation and cDNA synthesis

Only dietary LP (equivalent to current diploid diet formulation) and HP (“test” triploid diet formulation) treatments were assessed for gene expression. Vertebral sections at parr and smolt were manually cleaned of soft tissue and total RNA (totRNA) extracted according to the Trizol protocol (Invitrogen, UK), which was rehydrated in 12 μL of MilliQ water. Quality and concentration was validated with 1% agarose denaturing RNA gel electrophoresis and Nanodrop spectrophotometer ND-1000 (Labtech Int., East Sussex, UK) respectively. 5 μg totRNA was treated with DNase enzyme (DNA-free™: Applied biosystems, UK), concentrations revalidated with nanodrop and 1 μg of subsequent totRNA was reverse transcribed to cDNA using a high capacity reverse transcription kit without RNase inhibitor (Applied biosystems, UK). Final cDNA 20 μL reactions were diluted 1:10 in DNA/RNA free water to a total volume of 200 μL and 5 μL was used for each 20 μL qPCR reaction.

#### 2.4.2 Sequence information and primer design

Sequence specific primers for *mmp13*, *igf-Ir*, *alp* and *col1a* were based on registered sequence information in Atlantic salmon from the National Center for Biotechnology Information (NCBI) website (www.ncbi.nlm.nih.gov). All available sequence information was subjected to BLAST analysis against an Atlantic salmon transcriptome and genome (NCBI). Forward and reverse primers are outlined in Table 2 and were manufactured by MWG Eurofins Genomics (Ebersberg, Germany). Each primer pair was verified by PCR using MyTaq™ Mix (BIO-25041; Bioline) and produced a single band on a 1% agarose gel indicating a single product was available for sequencing. All products were subsequently purified using GeneJET PCR Purification Kit (KO701; ThermoFisher Scientific) and sequenced using Light Run sequencing service (GATC, Cologne). Resulting qPCR fragment sequences were verified with BLASTn and were suitably aligned with the target sequences.

**Table 2:** Primer sequences including housekeeping (HK) gene along with annealing temperatures and accession number used for qPCR.

Primer name	Forward	Reverse	Annealing temp (°C)	Reference	Accession no.
*qPCR*					
ALPQ1	ATCCTGCTCATCTGCTCCTGC	AGTATTCGTGCTGCCGTCACT	56	(Ytteborg et al., 2010)	Fj195609
Col1a1Q1	TGGTGAGCGTGGTGAGTCTG	TAGCTCCGGTGTTTCCAGCG	56	(Ytteborg et al., 2010)	Fj195608
Fgf23Q1	TCATCCAGCTCCGGCATAGC	AAGAACACGGTGCCACTGGA	57		XM_014153467.1 XM_014208327.1 XM_014208328.1
Igf1rQ1	AGCCACCTGAGGTCACTACG	ACATCCCGTCCGCTATCTCC	58	(Wargelius et al., 2005)	AY049954
Mmp13Q1	CCAACCCAGACAAGCCAGAT	GCTCTGAGAGTGGATACGCC	56	(Wargelius et al., 2010; Ytteborg et al., 2010)	NM_001140524.1

*HK gene*					
Elf-αq1	TCTGGAGACGCTGCTATTGTTG	GACTTTGTGACCTTGCCGCTTGAG	58	(Ytteborg et al., 2010)	NM_001123629.1

*Fgf23 sequence ID*					
Fgf23-1	TCGACTTGTGGATGCCCTTC	CACCCGGTTTCTCTTCTCCC	58	Designed on predicted sequence	XM_014153467.1 XM_014208327.1 XM_014208328.1
Fgf23-2	GGAGGGAGACTGAGGGGTCT	GAAACAAGAGAGCGCGTGGC	58	Designed on predicted sequence	XM_014153467.1 XM_014208327.1 XM_014208328.1

For *opn*, available nucleotide sequence information in all available fish species was used for T BLAST x in Atlantic salmon Expressed Sequence Tags (EST) and Whole Genome-Sequence contigs (WGS) databases and yielded no appropriate alignments. Nucleotide sequences were aligned by ClustalW and revealed little conservation in sequences between species. Hence, sequences were converted to the corresponding protein sequences and then aligned again by ClustalW, which revealed conserved regions with reference to brook trout: Region 1 = Protein 1–16 (136–183 nucleotide); Region 2 = Protein 150–184 (584–587 nucleotide); Region 3 = Protein 215–232 (780–683 nucleotide); Region 4 = protein 310–326 (1065–1113 nucleotide). Four primer pairs were designed in the corresponding nucleotide sequences in brook trout. Seven PCR reactions were carried out and sequenced using LIGHTrun™ Sequencing (GATC, Cologne, Germany) resulting in a 908 bp consensus sequence being generated for salmon. BLASTn results revealed 83% and 91% identity with brook and rainbow trout sequences. T BLAST x revealed a mean of 68%, protein identity with zebrafish. qPCR primers were then designed within this fragment (Table 2).

**Fig. 2:**
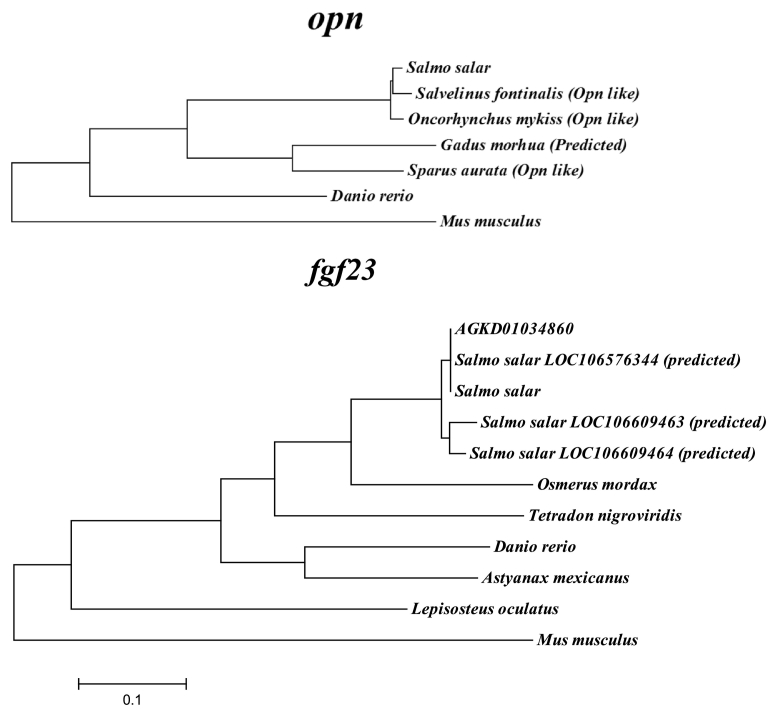
Molecular phylogenetic analysis by Maximum Likelihood method for *opn* (AF515708.1) and *fgf23* (NM_022657.4) with mouse (*Mus musculus*) provided as an outlier. Accession numbers for respective species sequences used are provided in Table 2. *Salmo salar fgf23* is on cloned *fgf23* fragment based on primers designed on WGS (product not registered on public database). The evolutionary history was inferred by using the Maximum Likelihood method based on the Tamura-Nei model (Tamura and Nei, 1993). The tree with the highest log likelihood (−4704.7297) is shown. Initial tree(s) for the heuristic search were obtained automatically by applying Neighbor-Join and BioNJ algorithms to a matrix of pairwise distances estimated using the Maximum Composite Likelihood (MCL) approach, and then selecting the topology with superior log likelihood value. The tree is drawn to scale, with branch lengths measured in the number of substitutions per site. The analysis involved 8 nucleotide sequences. Codon positions included were 1st + 2nd + 3rd + Noncoding. All positions containing gaps and missing data were eliminated. There were a total of 687 positions in the final dataset. Evolutionary analyses were conducted in MEGA6 (Tamura et al., 2013).

Sequence information was available for *fgf23* in a number of fish species and BLASTn revealed significant results in Atlantic salmon WGS (agkd01034860) and Atlantic salmon transcriptome predicted Fgf23-like sequences XM_014153467.1, XM_014208327.1 and XM_014208328.1. Phylogenetic trees were generated from available sequence information for *Opn* and *fgf23* (Fig. 2) Atlantic salmon WGS results were aligned to other available teleost sequences and displayed the highest identity to rainbow smelt (*Osmerus mordax*). Two sets of PCR primers for *fgf23* identification were designed based on the WGS sequence (agkd01034860) (Table 2), which was trimmed appropriately according to alignments in order to eliminate 5′ and 3′ regions and introns. PCR products generated from both primer sets were sequenced by LIGHTrun™ Sequencing (GATC, Cologne, Germany, qPCR primers were then designed within the sequenced product. Sequence information for all sets of primers is shown in Table 2.

#### 2.4.3 Real time qPCR

Absolute quantification qPCR assays were designed for genes described in Table 2 and performed in accordance with the Minimum Information for Publication of Quantitative Real-Time PCR Experiments (MIQE) guidelines (Bustin et al., 2009). Each qPCR assay was carried out in a 96 well plate using standardised plasmids of a known concentration with a linear range of 10^6^ and 10^2^ for all genes except the reference gene which was 10^7^–10^3^, which were generated based on appropriate PCR reactions for each cloned gene. Each 20 μL qPCR reaction consisted of primer pairs at a concentration of 6 pmol/μL 5 μL of cDNA, 3.8 μL DNA/RNA free H_2_O and 10 μL of Luminaris Colour HiGreen qPCR Master mix (Thermo scientific). The Luminaris Colour HiGreen qPCR Master mix was made up of: Hot Start Taq DNA Polymerase, UDG and dNTPs (also dUTP) in an optimized PCR buffer with a blue dye; supplied with 40× Yellow Sample Buffer and nuclease-free water. All assays were carried out in a Techne Quantica thermocycler (Techne, Quantica, Cambridge, UK) according to manufacturer's instructions under a thermocycling programme beginning with UDG pre-treatment of 50 °C for 2 min followed by 95 °C for 10 min (hot start). This was then followed by 40 cycles of 3 temperature steps; 95 °C for 15 s (melt) annealing at temperatures reported in Table 2 for 15 s and 72 °C for 30 s extension. This was followed by a temperature ramp from 60 to 90 °C for melt-curve analysis to verify that no primer–dimer artefacts were present and only one product was generated from each qPCR assay.

Cloning PCR products were purified using a Thermo genejet purification kit (Thermo Scientific, UK). Products were cloned using the pGEM®-T Vector System (Promega) and plasmids harvested using a GenElute™ Plasmid Miniprep Kit (Sigma Aldrich, UK). Resulting plasmids (forward and reverse for each, n = 2) were individually sequenced via SUPREMErun™ sequencing (GATC, Cologne Germany) to confirm identity. Plasmids were then linearised by enzyme digest (*Hin*cII; NewEngland Biosciences) and standards for qPCR assays were generated using a serial dilution from 10^8^ copies to 10 copies of each gene investigated. Efficacy for all assays was between 1.95 and 2. All samples were run in duplicate and each assay contained non-template controls. Results from vertebral cDNAs were normalised by relating expression data to the reference gene (*Elf-α*) as described in Ytteborg et al. (2010). Normalisation of diploid and triploid gene expression was carried out in a ploidy specific manor in order to account for potential differences in gene expression resulting from the existence of a third allele present in triploid individuals. Stability of the reference gene was verified, as no significant difference in expression was observed between sample points.

### 2.5 Calculations

Condition factor (K) was calculated as: *W* × (*L*^3^)^−1^ × 100. Specific growth rate (SGR) was calculated as: SGR = (e^g^ − 1) × 100, where g = [ln(W_2_) − ln(W_1_)] × (t_2_ − t_1_)^−1^, where W_2_ and W_1_ are the weight at t_2_ and t_1_ respectively, with *t* denoting time. Due to technical constraints on the system, waste feed collection was not possible, and thus biological FCR (bFCR) could not be calculated, although waste pellets were evident on all occasions on daily tank flushing ensuring station was achieved.

### 2.6 Statistical analyses

All data were analysed and compared using the R language (R Core Team, 2013) and significance was accepted at 5% (*p* < 0.05). Percentage datasets were arcsine transformed prior to analysis. Results are reported as mean ± standard error of the mean. Datasets confirmed to be normal and homogeneous were analysed using the *lme* function in the *nlme* package for Two-Way-ANOVA with replicates nested within ploidy and diet. Datasets including life stage interaction (minerals and gene expression) were analysed with a Three-Way ANOVA with replicates nested within ploidy and diet. Where no significant three-way interaction was observed Two-Way ANOVAs were performed for each life stage as it was necessary to retain individual life stages. Post-hoc analysis was performed using the *glht* function for Tukey's multiple comparison in the *multcomp* package where significant differences were observed. Radiography deformity datasets showed a negative binomial distribution confirmed with the function *odTest* in the *pscl* package hence a generalised linear model with over-dispersion was performed using the *glm.nb* function in the *MASS* package. As well as deviance analysis, post-hoc analysis was performed using the *glht* function for Tukey's multiple comparison in the *multcomp* package.

## 3 Results

### 3.1 Cumulative mortality and growth

Mortality from egg receipt to first feeding did not differ significantly between ploidies (diploids: 4.41 ± 0.98%; triploids: 5.06 ± 0.94%). Cumulative mortality from first feeding to smolt did not differ significantly between ploidies or diet (data not shown), although there was a trend for decreasing mortality with increasing dietary P inclusion level in both ploidies (diploids: LP 4.29 ± 0.19%; MP 3.46 ± 0.20%; HP 3.29 ± 1.21%; triploids: LP 4.13 ± 0.38%; MP 3.77 ± 0.07%; HP 3.59 ± 0.86%). A 0.5 to 1.1% increase in cumulative mortality was observed in triploids and diploids in LP diets respectively in days 1–3 post-vaccination, while MP and HP diets showed no such peak during the same period.

Triploids started first feeding at an 11.7% and 3% significantly smaller weight and length, respectively, compared to diploids, although K factor did not differ between ploidies (Table 3). From first feeding to parr, diploids showed a significantly reduced SGRwt than triploids (2.62 ± 0.04 vs. 2.76 ± 0.10% day^−1^, *p* = 0.0001), such that weight at parr did not differ significantly between ploidies except for triploid fish fed HP which were significantly heavier than their respective diploids, and larger than all other treatments except diploid LP (Table 3). Diploid LP was significantly heavier than MP but not HP. Parr length was not affected by ploidy, but was affected by diet and an interaction with ploidy, such that triploid HP were significantly longer than all treatments other than diploid LP. In diploids, fish fed MP were significantly shorter than diploid LP but not HP. By contrast, K factor at parr did not differ between ploidies, but was significantly affected by diet, being lower in HP than LP and MP groups in both ploidies.

**Table 3:** Performance summary for diploid and triploid fish fed on low (LP), medium (MP) and high (HP) dietary P inclusion (mean ± SEM, n = 2). Superscripts denote significant differences between dietary treatment and ploidy within stage of development (newly hatched, parr and smolt) (*p* < 0.05; Two-Way ANOVA).

	LP		MP		HP		Significance		
	Dip	Trip	Dip	Trip	Dip	Trip	Ploidy	Diet	P × D
First feed weight (g)	0.17^a^ ± 0.01	0.15^b^ ± 0.01	0.17^a^ ± 0.01	0.15^b^ ± 0.01	0.17^a^ ± 0.01	0.15^b^ ± 0.01	**0.003**	n/a	n/a
First feed length (mm)	28.1^a^ ± 0.2	27.3^b^ ± 0.8	28.1^a^ ± 0.2	27.3^b^ ± 0.8	28.1^a^ ± 0.2	27.3^b^ ± 0.8	**0.0001**	n/a	n/a
First feed condition (K)	0.66 ± 0.01	0.65 ± 0.01	0.66 ± 0.01	0.65 ± 0.01	0.66 ± 0.01	0.65 ± 0.01	0.420	n/a	n/a
Parr weight (g)	5.9^ab^ ± 0.4	5.6^bc^ ± 0.5	5.0^c^ ± 0.6	5.1^c^ ± 0.5	5.5^bc^ ± 0.4	6.4^a^ ± 0.5	**0.001**	**0.025**	0.169
Parr length (mm)	80.4^ab^ ± 1.1	79.2^bc^ ± 1.1	76.3^c^ ± 1.2	76.4^c^ ± 1.1	79.1^bc^ ± 1.2	83.4^a^ ± 1.2	0.123	**0.0001**	**0.005**
Parr condition (K)	1.09^a^ ± 0.01	1.10^a^ ± 0.01	1.08^ab^ ± 0.01	1.10^a^ ± 0.01	1.07^b^ ± 0.01	1.07^b^ ± 0.01	0.191	**0.0001**	0.149
First feed - parr SGRwt	2.66^abc^ ± 0.03	2.79^ab^ ± 0.10	2.58^c^ ± 0.11	2.65^bc^ ± 0.05	2.61^bc^ ± 0.04	2.84^a^ ± 0.04	**0.0001**	**0.030**	0.159
Smolt weight (g)	46.2^ab^ ± 1.1	48.6^a^ ± 0.9	39.4^c^ ± 0.7	46.5^ab^ ± 0.7	41.3^c^ ± 0.9	45.6^b^ ± 0.8	**0.0001**	**0.0001**	**0.008**
Smolt length (mm)	163.8^b^ ± 1.3	168.3^a^ ± 1.0	154.9^c^ ± 1.0	165.8^ab^ ± 0.9	157.9^c^ ± 1.1	163.9^b^ ± 1.1	**0.0001**	**0.0001**	**0.005**
Smolt condition (K)	1.04^ab^ ± 0.01	1.01^c^ ± 0.01	1.06^a^ ± 0.01	1.02^bc^ ± 0.01	1.04^a^ ± 0.01	1.03^abc^ ± 0.01	**0.0001**	0.195	0.129
Parr-smolt SGRwt	0.82 ± 0.05	0.85 ± 0.01	0.84 ± 0.08	0.84 ± 0.07	0.80 ± 0.02	0.78 ± 0.02	0.821	0.140	0.582
First feed – smolt SGRwt	1.45^bc^ ± 0.02	1.52^a^ ± 0.03	1.44^bc^ ± 0.01	1.46^abc^ ± 0.03	1.42^c^ ± 0.01	1.48^ab^ ± 0.01	**0.0001**	**0.030**	0.111

n/a: not applicable.

Significant values (*p* < 0.05) are highlighted in bold.

Final smolt weight and length were both affected by ploidy and diet, and with an interaction between the two, such that triploids fed LP diet had a higher final weight and length than HP, and MP diet smolt weight intermediary (Table 3). In diploids, LP achieved a significantly higher final weight and length than MP and HP diets. Smolt K factor was not affected by diet, but was significantly affected by ploidy, being lower in triploids than diploids in LP and MP diets but not HP. From parr to smolt, SGRwt did not differ significantly between ploidies.

In terms of overall performance, total SGR from first-feeding to smolt differed significantly between ploidies (diploids: 1.43 ± 0.02 vs. triploids: 1.49 ± 0.02% day^−1^, *p* < 0.0136), and diets (LP: 1.50 ± 0.03, MP: 1.47 ± 0.03, HP: 1.46 ± 0.02).

### 3.2 Vertebral assessment

#### 3.2.1 Whole mount staining

**Fig. 3:**
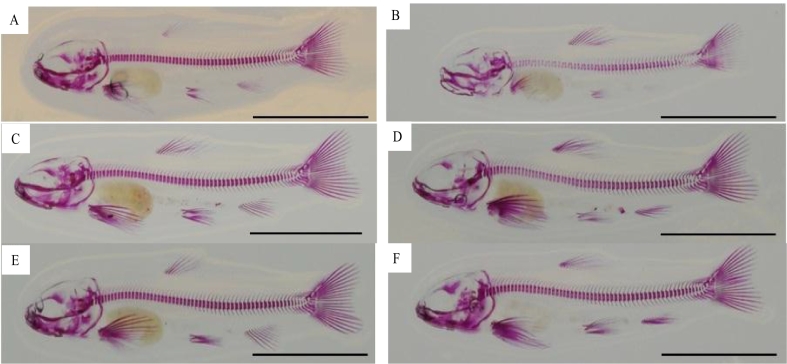
Whole mount staining for calcium 3 weeks post-first feeding (216 °D post-feeding) of diploid (A, C, E) and triploid (B, D, F) fish fed LP (A, B), MP (C, D) and HP (E, F) dietary P inclusion. Scale bar = 10 mm.

No observable differences were found in cartilage levels indicated by Alcian blue staining between diet and ploidies in fry stages. However, there were higher amounts of observable cartilage between 3 and 8 weeks post-first feeding in all treatments. Calcification, indicated by Ca staining with alizarin red, showed visually comparable levels of ossification irrespective of dietary treatments in diploids at 3 weeks post-feeding (Fig. 3 A, C, E). By contrast, increased levels of ossification of the vertebral column were visually apparent in both MP and HP diets compared to LP in triploids (Fig. 3 B, D, F).

Individuals were not large enough for accurate quantifiable image analysis measurements until 8 weeks post-first feeding (Table 4). Significant interactions of diet and ploidy on mean vertebral area (mm^2^) were only found in the caudal area (R3 & R4) where vertebral areas were significantly lower in triploid LP relative to all other treatments.

**Table 4:** a) Vertebral area (mm^2^) based on whole mount Ca staining at 0.5 g (8 weeks post-first feeding); and b) externally observable jaw and vertebral deformities at smolt (mean ± SEM, n = 2) for diploid and triploid fish fed on low (LP), medium (MP) and high (HP) dietary P inclusion. Different uppercase superscript letters denote significant differences in vertebral area between ploidy & diet within a given spinal region (R1–R4) in fry; while lowercase superscripts denote significant differences between ploidy and diet within externally visible jaw and vertebral deformity at smolt (*p* < 0.05; Two-Way ANOVA/nbGLM & Tukey's post hoc).

	LP		MP		HP		Significance		
	Dip	Trip	Dip	Trip	Dip	Trip	Ploidy	Diet	P × D
a) Vertebral area (mm^2^) - 0.5 g fry⁎									
Cranial-trunk (R1)	0.50 ± 0.02	0.45 ± 0.01	0.53 ± 0.05	0.55 ± 0.01	0.59 ± 0.02	0.53 ± 0.01	0.3	**0.04**	0.4
Caudal-trunk (R2)	0.43 ± 0.01	0.39 ± 0.01	0.44 ± 0.08	0.51 ± 0.03	0.50 ± 0.03	0.47 ± 0.01	0.9	**0.009**	0.1
Tail region (R3)	0.73 ± 0.02^A^	0.59 ± 0.02^B^	0.75 ± 0.07^A^	0.72 ± 0.03^A^	0.77 ± 0.01^A^	0.77 ± 0.05^A^	**0.02**	**0.0008**	**0.04**
Tail fin (R4)	0.34 ± 0.01^A^	0.21 ± 0.04^B^	0.39 ± 0.04^A^	0.39 ± 0.01^A^	0.40 ± 0.01^A^	0.40 ± 0.01^A^	**0.02**	**0.0001**	**0.005**
b) Visible deformity – smolt (%)†									
Jaw	0.6 ± 0.2^bc^	7.7 ± 0.3^a^	0.0 ± 0.0 ^c^	0.0 ± 0.0^c^	0.1 ± 0.1^bc^	1.2 ± 0.5^b^	**0.0001**	**0.0001**	**0.002**
Vertebral	0.0 ± 0.0^b^	0.0 ± 0.0^b^	2.0 ± 0.0^a^	2.0 ± 0.0^a^	0.0 ± 0.0^b^	0.0 ± 0.0^b^	0.9	**0.0001**	0.9

ns = non-significant.

Significant values (*p* < 0.05) are highlighted in bold.

⁎ 3 vertebrae/5 individuals/tank (n = 2, 10/treatment).

† Percentage deformed of entire population per tank assessed at smolt (n = 385–440 fish/tank).

#### 3.2.2 External assessment

Entire population (385–440 fish/tank) assessment upon termination of the trial at smolt revealed a significantly higher number of individuals with observable jaw malformations, predominantly twisted lower jaws, in triploid LP (7.7 ± 0.3%) than other treatments (Table 4). However, the proportionate increase in jaw deformities associated with triploidy was the same in LP and HP, in that prevalence of 7.7% and 1.2% were 13- and 12-fold higher than diploid values of 0.6% and 0.1% for LP and HP respectively. Frequencies of external skeletal malformations were overall low for all treatments, but were significantly higher in MP diets than LP and HP irrespective of ploidy.

#### 3.2.3 Radiological assessment

**Fig. 4:**
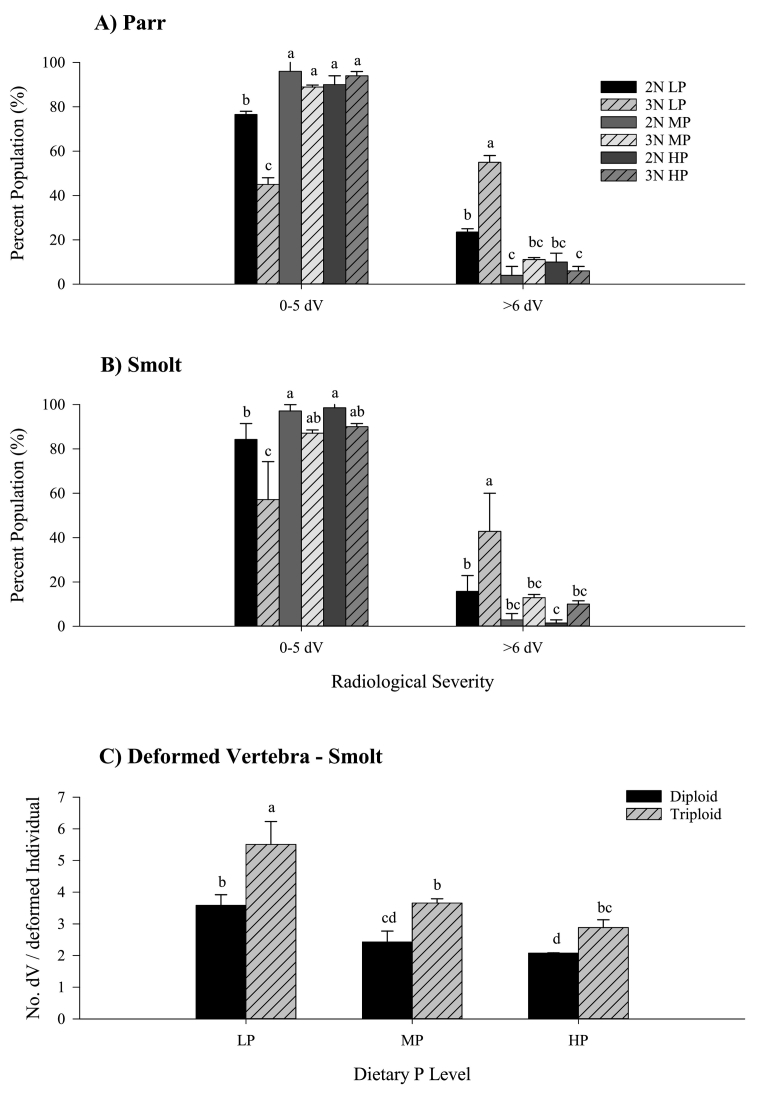
Percent population (%) classified according to severity of detectable radiological spinal malformation (none–mild: 0–5 dV; moderate–severe ≥6 dV) (mean ± SEM, n = 2) in A) parr (50/tank) and B) smolt (35/tank) for diploid and triploid salmon fed increasing dietary P inclusion (LP, MP and HP); and C) number of deformed vertebra per deformed individual assessed at smolt. Lowercase superscripts denote significant differences between ploidy and diet within each spinal region for a given spinal severity classification (0–5 dV and ≥6 dV) within each life stage (fig A parr; fig B smolt), and between diet and ploidy for no. dV affected (fig C) (*p* < 0.05; Two-Way ANOVA & Tukey's post hoc).

Total number of vertebrae (mean 59.0 ± 0.1) was not significantly affected by ploidy or diet. At parr stage, fish fed MP and HP diets in both ploidy had a significantly greater percentage of the population expressing none to mild spinal deformities (0–5 dV) than fish fed LP diet (Fig. 4A). There was also a significant effect of ploidy in the LP diet, with triploids having a significantly lower percentage of the population showing 0–5 dV than diploids. Conversely, triploid LP had a significantly higher percentage of the population expressing more severe spinal deformities (≥6 dV) than all other treatments. Diploids had a significantly higher prevalence in LP than MP but not HP diets. No significant difference between ploidies was apparent for MP and HP diets in individuals with ≥6 dV. Generally the same diet-ploidy pattern observed at parr assessment was observed at smolt (Fig. 4B). Triploid MP and HP had a comparable percentage of the population expressing 0–5 dV as observed in diploid LP, and the same pattern was present in more severely affected (≥6 dV) individuals. In both ploidy, number of dV per deformed individual decreased significantly from LP to MP, but was not improved significantly in the HP diet (Fig. 4C). Within each diet, triploids consistently had a significantly higher number of deformed vertebrae than their corresponding diploids.

**Fig. 5:**
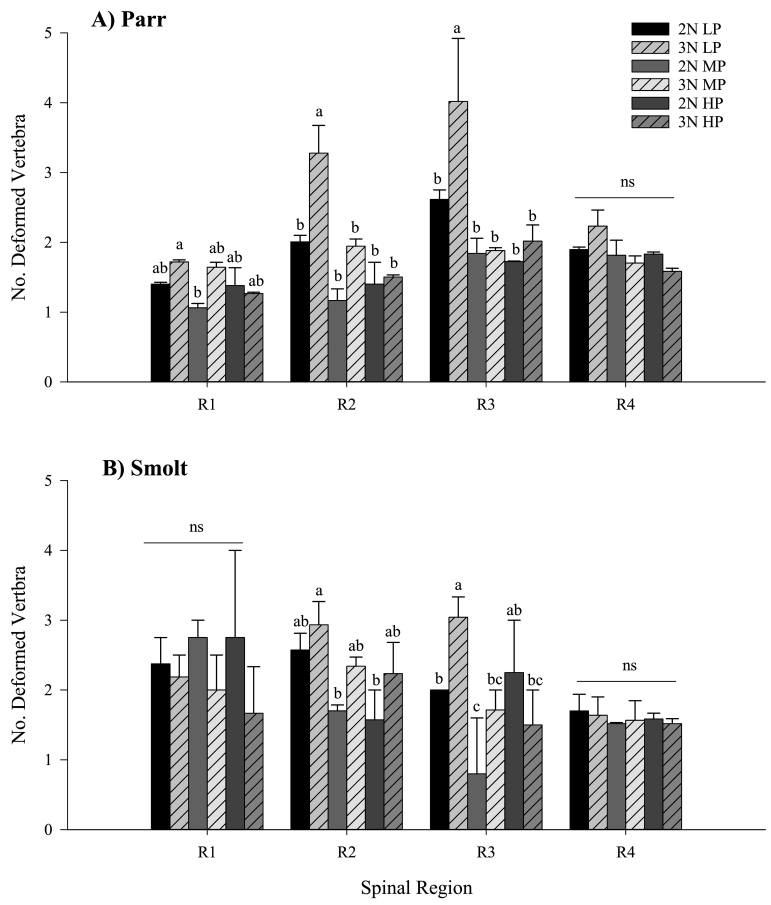
Number of deformed vertebra per deformed individual per spinal region (mean no. ± SEM, n = 2) in A) parr (50/tank)and B) smolt (35/tank) for diploid and triploid salmon fed increasing dietary P inclusion (LP, MP and HP). Lowercase superscripts denote significant differences between ploidy and diet within each respective spinal region (*p* < 0.05; Two-Way ANOVA & Tukey's post hoc). Regions R1 (v1–8), R2 (v9–30), R3 (v31–49), and R4 (v50–58/59/60).

Overall, the majority of vertebral malformations were prevalent in the central (v27–31) and tail region (v52–57) in both parr and smolt (Supplementary file A). Both diet and ploidy significantly affected the number of deformed vertebra (dV) per deformed individual in spinal regions R1–R3 in parr, but only R2 and R3 at smolt (Fig. 5A–B). Occurrence of deformed vertebra was only affected by life stage in R1 and R2, with a significant increase from parr to smolt (R1, *p* = 0.001; R2, *p* = 0.01). There was no overall significant difference in R3 & R4 from parr to smolt (R3, *p* = 0.346; R4, *p* = 0.066). In parr, no effect of ploidy was observed in R1, with only triploid LP having a higher no. of dV than diploid MP. In R2 and R3, triploid LP had significantly higher no. dV than all other treatments. At smolt, triploid LP had a significantly higher no. of dV in R3 than their respective diploid group, and all other treatments with the exception of diploid HP.

Within deformed individuals, fifteen pathology types were observed in parr and fourteen pathology types at smolt. Type 10 deformities (widely spaced & undersized vertebral bodies) were only recorded in parr, and only in triploid LP (5%) and MP diets (1%). All other pathologies recorded in parr were evident in smolts, and subsequently, pathologies were reclassified into six main types (Table 5). The most prevalent pathology recorded in all treatments were symmetry shifts (Type 17, and mostly Type 19) accounting for approximately 50% of all pathologies recorded, and predominantly located in caudal trunk (R2) and tail fin (R4). In both ploidies, there was a progressive decrease in occurrence of symmetry shifts within increasing dietary P inclusion level, although within each diet, triploids presented approximately twice the number recorded relative to their diploid group. Radio-translucent/opaque vertebral bodies accounted on average for approximately 24% of remaining total pathology in all groups. Again triploids presented greater no. of affected vertebrae than their diploid counterparts across all dietary groups. LP diets showed greatest numbers affected, with a marked reduction in MP and HP dietary groups relative to LP diets. Occurrence of decreased inter-vertebral spacing (Type 1) decreased with increasing dietary P level in diploids, and was predominantly observed in the cranial trunk (R1) and cranial trunk (R2). By contrast, type 1 pathology was principally observed in the trunk region (R2 & R3) in triploids, and highest in MP diets. Compression pathologies (principally Type 2, homogenous compression, and Type 5, one-sided) were comparable between diploid LP and MP diets, and were principally located in the tail fin (R4), and decreased in the HP diet. In triploids, compression pathology was predominantly located in the caudal trunk (R2), and generally showed a decrease with increasing dietary P level from LP to MP. Occurrence of fusion (Type 6–8) pathology was generally low in both ploidies at smolt (~4% of total pathology recorded), and was generally comparable between LP and MP diets, but decreased in HP diets. Of fusion pathologies recorded, only triploids in LP and MP diets presented Type 8 (fusion centre) principally located in the caudal trunk (R2) and tail fin (R4) respectively.

**Fig. 6:**
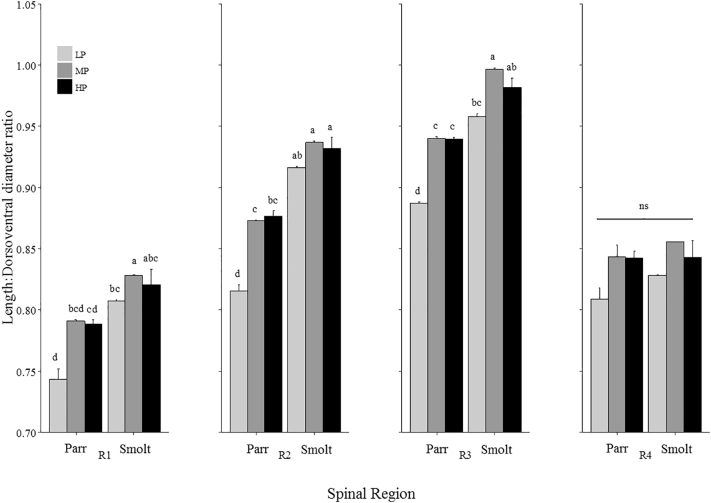
Mean length: dorso-ventral diameters ratios (mean ± SEM, n = 2, 10 fish/treatment) of vertebrae taken from x-rays at parr (5 g) and smolt (45 g) for pooled diploid and triploid fish fed differing dietary P inclusion levels (LP, MP and HP). Regional values were calculated as the mean L:H ratio per region R1 (v1–8), R2 (v9–30), R3 (v31–49), and R4 (v50–58/59/60) for each individual. Superscripts denote significant differences within each spinal region between dietary treatments and stage (*p* < 0.05; Two-Way ANOVA & Tukey's post hoc).

**Table 5:** Types of vertebral pathology (total number, classified according to Witten et al. (2009) at smolt.

Pathology type	LP		MP		HP	
	Dip^b^	Trip^b^	Dip^b^	Trip^b^	Dip^b^	Trip^b^
Decreased intervertebral space	26	25	5	46	4	10
Compression	22	43	25	12	16	19
Fusion	6	12	10	11	3	2
Elongation	1	0	0	0	0	4
Radio-translucent/opaque	39	139	8	29	10	37
Symmetry shift	82	157	61	100	50	91
∑^a^	176	376	109	198	83	163

a Total no. vertebrae affected.

b No. vertebra examined (2N LP = 4154; 3N LP = 4072; 2N MP = 4093; 3N MP = 4132; 2N HP = 4094; 3N HP = 3954).

Individual length: dorso-ventral diameter profiles for each ploidy and diet are provided in Supplementary file B. Although overall, triploids had significantly increased length: dorso-ventral diameter (L:H) ratios compared to diploids for each region (dip and trip respectively; R1: 0.78 & 0.82 ± 0.03, *p* = 0.0003; R2: 0.88 & 0.91 ± 0.04, *p* = 0.0008; R3: 0.94 & 0.96 ± 0.03, *p* = 0.006; R4: 0.82 & 0.85 ± 0.03, *p* = 0.006), there was no significant effect of ploidy with diet or life stage interactions. However, a consistent significant effect of life stage within diet was observed with the exception of Region 4 (R1: *p* = 0.04; R2: *p* = 0.0005; R3: *p* = 0.01; R4: *p* = 0.5; Fig. 6). L:H ratios were significantly greater at smolt compared to parr for LP in R1 and all dietary treatments in R2 and R3. L:H ratios were significantly shorter in LP parr compared to MP and HP parr for R2–3. There was no significant difference in L:H ratios between dietary treatments at smolt for R1–2, however, MP had significantly higher L:H ratio compared to LP in R3.

### 3.3 Whole body mineral concentration

Final smolt whole body phosphorous (P) and vanadium (V) composition was only significantly affected by life stage, while ploidy and diet only affected calcium (Ca), Ca:P ratio and zinc (Zn) (Table 6). Whole body P content significantly increased from parr to smolt in both ploidies fed LP, and triploid HP. Lowest P content was found in triploid LP at parr, while triploid HP smolts had significantly higher body P than all other treatments at smolt. At parr, whole body Ca was significantly (*p* = 0.04) higher in diploids than triploids (4029 vs. 3733 μg/mg), and significantly (*p* = 0.0001) increased from LP (3143 ± 130 μg/mg) to MP diet (4303 ± 127 μg/mg), but did not increase further in the HP diet (4198 ± 122 μg/mg). Final smolt Ca content did not differ between ploidies or diet in LP and MP treatments, while a significantly higher Ca content was observed in triploids relative to diploids fed the HP diet. At parr whole body Ca:P ratio only differed significantly (*p* = 0.03) between ploidies (Diploids: 1.05 ± 0.07 vs. Triploids: 1.00 ± 0.1) but not at smolt. Similarly, diet affected whole body Ca:P ratio only at parr, and was independent of ploidy, with Ca:P ratio significantly (*p* = 0.0001) increasing between LP (0.93 ± 0.06) and MP diet (1.08 ± 0.01) but no further increase in the HP diet (1.07 ± 0.02). V concentrations generally increased from parr to smolt, and with increasing dietary P inclusion level. Lowest levels were observed in triploid LP parr, while highest V concentrations were observed in triploid HP at smolt, significantly higher than all treatments at parr. No significant differences in Zn content were observed at parr. At smolt, Zn content was significantly higher triploids relative to diploids fed HP, while no other effects between ploidy and diet were evident.

**Table 6:** Whole body mineral composition (μg/mg, mean ± SEM, n = 2, 15 fish/tank) at parr and smolt stages for diploid and triploid Atlantic salmon fed on low (LP), medium (MP) and high (HP) dietary P inclusion.

Whole body mineral composition†		LP		MP		HP		Significance				
		Dip	Trip	Dip	Trip	Dip	Trip	Ploidy	Diet	Stage	P × D	P × D × S
Phosphorous	Parr	3460 ± 44^cd^	3259 ± 129^d^	3985 ± 17^bc^	3938 ± 84^bcd^	4014 ± 52^bc^	3864 ± 43^bcd^	0.3	**0.0004**	**0.0001**		**0.02**
	Smolt	4323 ± 92^b^	4421 ± 180^b^	4254 ± 272^b^	4132 ± 169^bc^	4193 ± 206^b^	5119 ± 21^a^					
Calcium	Parr	3368 ± 108	2917 ± 221	4377 ± 44	4229 ± 160	4342 ± 92	4054 ± 131	**0.04**	**0.0001**	NA	0.7	0.08
	Smolt	4601 ± 121^AB^	4661 ± 275^AB^	4296 ± 453^B^	4193 ± 157^B^	4331 ± 256^B^	5425 ± 206^A^	0.09	0.05	NA	**0.04**	
Ca:P Ratio	Parr	0.97 ± 0.02	0.89 ± 0.03	1.09 ± 0.02	1.07 ± 0.02	1.08 ± 0.01	1.05 ± 0.02	**0.03**	**0.0001**	NA	0.5	0.9
	Smolt	1.06 ± 0.00	1.05 ± 0.02	1.01 ± 0.04	1.02 ± 0.01	1.03 ± 0.01	1.06 ± 0.03	0.6	0.3	NA	0.7	
Vanadium	Parr	0.023 ± 0.000^cd^	0.022 ± 0.001^d^	0.027 ± 0.000^bcd^	0.025 ± 0.000^cd^	0.039 ± 0.000^b^	0.033 ± 0.000^bcd^	0.07	**0.0001**	**0.0001**	NA	**0.003**
	Smolt	0.031 ± 0.003^cd^	0.041 ± 0.007^b^	0.040 ± 0.004^b^	0.036 ± 0.005^bc^	0.036 ± 0.011^bc^	0.058 ± 0.009^a^				NA	
Zinc	Parr	44.03 ± 2.67	44.93 ± 1.86	43.95 ± 1.08	42.42 ± 0.35	42.13 ± 1.10	39.98 ± 1.07	0.5	0.2	NA	0.7	0.06
	Smolt	37.90 ± 0.71^AB^	41.27 ± 3.75^AB^	40.10 ± 1.81^AB^	36.88 ± 1.07^AB^	36.42 ± 1.63^B^	45.19 ± 1.44^A^	0.08	0.5	NA	**0.02**	

^a, b, c, d^Mean values with different lowercase superscript letters are significantly different between ploidy (P) × diet (D) × life stage (S) (*p* < 0.05; Three-Way ANOVA & Tukey's post hoc).

^A, B, C, D^Mean values with different uppercase superscript letters are significantly different between ploidy (P) × diet (D) within a life stage (Parr or smolt) (*p* < 0.05; Two-Way ANOVA & Tukey's post hoc).

Significant values (*p* < 0.05) are highlighted in bold.

† 5 fish pooled in triplicate per tank (30 fish/treatment).

No significant differences were observed in smolt bone mineral percentage (BM%, data not shown) in vertebral columns between diet or ploidies (42.5 ± 1.6%, *p* = 0.300). Triploids did however, have a significantly lower jaw BM% compared to diploids at smolt (26.7 ± 1.5% vs. 29.4 ± 2.2%, *p* = 0.01) but did not differ between diet (*p* = 0.07).

**Fig. 7:**
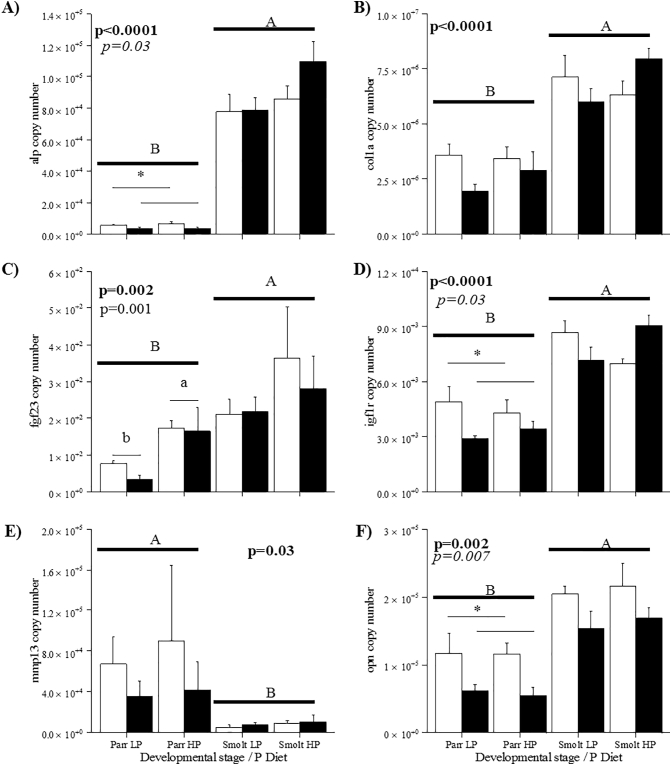
Gene expression (copy no.) of *alp*, *col1a*, *fgf23*, *igf1r*, *mmp13* and *opn* for diploids (white) and triploids (black) fed LP and HP diets (n = 2, 6 individuals/replicate) at parr and smolt. Capital subscripts denote significant differences between life stages (*p* < 0.05; Three-Way ANOVA), asterisks denote significant differences between ploidy (*p* < 0.05; Two-Way ANOVA) and lower case superscripts denote differences between diet (*p* < 0.05; Two-Way ANOVA).

### 3.4 Gene identification and expression of key bone markers

Sequencing of larger *Fgf23* fragment resulted in the generation of a 752 bp fragment showing 100, 95 and 96% nucleotide identity to *Salmo salar Fgf23* like predicted sequences XM_014153467.1, XM_014208327.1 and XM_014208328.1 respectively. With respect to *Osmerus mordax* and *Danio rerio* nucleotide identity is 77 and 74% respectively (Fig. 2). The sequenced product covers 92% of the coding region and runs 32 bp into the 3′ region in comparison to XM_014153467.1. The translated protein sequence contains FGF region, receptor interaction and heparin binding sites (see Supplementary file C). There was no significant interaction for ploidy, diet and life stage although there was a significant effect of life stage on gene expression for all genes (Fig. 7A–F), in which significantly lower mRNA expression levels were observed in parr than at smolt with the exception of *mmp13*, for which there was a significant increase in mRNA expression levels (Fig. 7E). Significantly higher expression levels of *alp* were observed in diploid than triploid parr (Fig. 7A). For *fgf23* both diploid and triploid HP parr had significantly higher expression than LP diploids and triploids (Fig. 7C). Significantly higher expression levels of *igf1r* (Fig. 7D) and *opn* (Fig. 7F) were observed in diploid parr compared to triploid parr, but this did not differ between diets. Furthermore, no significant differences between treatments were observed within the smolt stages.

## 4 Discussion

Results from this study further support the importance of increased dietary P in order to improve skeletal health of triploid Atlantic salmon during freshwater ongrowing, with greatest impact in the earlier life stages. Feeding a higher dietary P (7.7 to 9.7 g available P kg^−1^) reduced the incidence of radiologically detectable skeletal malformations and side twisted jaw pathology, but did reduce growth beyond the parr stage at the highest inclusion level.

Mortality rate in the current study from first-feeding to smolt did not differ statistically between ploidies or dietary P inclusion level in the diet, which is in accordance with most recent studies in triploids (Fjelldal and Hansen, 2010; Taylor et al., 2012; Taylor et al., 2013) although some studies, including recent, have reported higher mortalities (O'Flynn et al., 1997; Cotter et al., 2002; Amoroso et al., 2016a). Such improvements in triploid survival in more recent studies may be attributed to a better understanding of factors affecting triploid performance including improved egg quality (Taylor et al., 2011) and a recommendation for lower egg incubation temperatures (Fraser et al., 2014c). However, although non-significant, there was a trend for increasing survival with increasing dietary P inclusion level (4.9, 7.7, 9.7 g available P kg^−1^) which concurs with observations reported in a similar dietary P trial (4.0, 6.0, 12.0 g available P kg^−1^) in triploids by Fjelldal et al. (2015). In the current study, a small increase in mortality also occurred in both ploidies between 1 and 3 days post-vaccination in the LP diet but not MP and HP groups, suggesting there may be an interaction between dietary P and post-vaccination losses. Similarly, Fjelldal et al. (2015) also reported that mortality rate was highest in LP diets (4.0 g available P kg^−1^) in the period from 26th Aug (post-vaccination) to sea transfer (30th Sept) under an S0+ regime and hypothesised this may reflect severe signs of P deficiency rather than vaccination. However, Asgard and Shearer (1997) reported that while weight gain was somewhat affected by dietary P level (4.4 to 25 g kg^−1^ digestible P) in juvenile Atlantic salmon, mortality did not differ. Several studies have examined the interaction of ploidy on vaccination response (Fraser et al., 2012b; Fraser et al., 2014a; Fraser et al., 2014c), however, none reported whether survival differed between ploidies, and dietary P levels were not stated. Given the apparent interaction between low dietary P and increased mortality in periods post-vaccination in the current study and that of Fjelldal et al. (2015), further studies are warranted to elucidate the cause of this apparent effect, particularly given that triploids appear to be more prone to post-vaccination side-effects when fed commercial diploid feeds in freshwater (Fraser et al., 2014b).

As reported in other freshwater studies, triploids started first-feeding at a significantly smaller weight than diploids (Leclercq et al., 2011; Taylor et al., 2011; Taylor et al., 2012; Fjelldal et al., 2015). In the period from first-feeding to parr stage (~6 g, 1200 °days post-feeding), triploids showed higher SGRs than diploids, and growth effects were further accentuated in the HP diet, which concurs with growth effects reported by Fjelldal et al. (2015) during a similar developmental period (up to ~9 g, 1500 °days) under an S0+ regime. K factor in parr was affected by diet rather than ploidy, with HP diets having a lower K than LP and MP diets, potentially indicating increased skeletal mineral deposition and growth at this stage. Subsequently, from parr to smolt, SGR was not affected by dietary P level in either ploidy, although there was a trend towards reduced weight gain with increased dietary P such that triploids fed LP diet had a higher final smolt weight than HP diets, with MP intermediary to both. By contrast, diploid growth was impeded by increasing dietary P level, such that MP and HP diploids had a smaller final smolt weight than LP diploids and all triploid groups. These observations on growth rate again concur with results of Fjelldal et al. (2015), and further support the fact that triploids appear to require more available P than diploids in early freshwater life stages to support optimal growth. The inhibitory effect of feeding higher dietary P (13.9 vs. 7.6 g total P kg^−1^) on triploid growth at later life stages in freshwater (diets applied to 45–80 g) was also reported in an earlier study by Burke et al. (2010). Lower weights in diploid parr and smolt and triploid smolt fed higher P may be related to a reduced net intake of other nutrients or shifts in gut integrity as a result of excess dietary P (Boersma and Elser, 2006). Collectively, the delayed inhibitory effect of high P in triploid smolts would indicate that dietary requirements may indeed be higher for growth than diploids in the fry and parr stages. In support, triploids also showed a lower K factor than diploids at smolt stage as reported in numerous other studies (Fjelldal and Hansen, 2010; Taylor et al., 2012; Taylor et al., 2013; Fraser et al., 2013; Fjelldal et al., 2015), which supports the hypothesis of a differential pattern in somatic and skeletal growth between ploidies, although a lower total body lipid content in triploids cannot be discounted (Herbinger and Friarrs, 1991). A lower K factor for a given body weight could reflect a more rapid increase in skeletal length, which subsequently would incur a higher mineral requirement to support optimal bone mineralisation and development. Collectively, results would suggest that increasing dietary P alone does not enhance growth beyond the parr stage in triploids, and dietary P may play a more important role in supporting skeletal health.

A significantly higher incidence of visible jaw malformations, predominantly side twisting of the lower jaw, was observed in triploids fed the LP diet at smolt, although this was not reflected in bone mineral content which was comparable to MP and HP diets. However, although jaw pathology in both ploidies was reduced by HP diets, proportionally, prevalence in both LP and HP diets was still 12–13 time greater in triploids than their respective diploid siblings. Jaw malformations in diploids have been linked to available P deficiency in conjunction with higher water temperatures (20 °C; Roberts et al., 2001). Very low prevalence of jaw malformations in diploids in the current trial supports the concept that dietary P requirement is lower than for triploids. Fjelldal et al. (2015) found no jaw malformations in smolts (~56–62 g) fed 6.0–12.0 g available P kg^−1^ of which the MP and HP diets in the current study fall within the range suggested, while a large percentage in both diploids and triploids (79–89%) when fed 4.0 g available P kg^−1^. Available P was ~1 g lower than that of this study (4.9 g available P kg^−1^) which may explain the considerably higher prevalence of jaw deformity in the former study. Based on the results of the current study and Fjelldal et al. (2015), it could be suggested dietary P requirements for optimal jaw development in triploids appears to be between 6.0 and 7.7 g available P kg^−1^. However, Fraser et al. (2014c), showed that prevalence of jaw pathology increased in triploids, in addition to spinal deformity, when fed a commercial diploid diet (dietary P level not stated) following increasing egg incubation temperature (6, 8 and 10 °C from fertilisation to 1st feed). As such, although HP diets reduced prevalence of jaw deformity in the current study, it may be that sub-optimal egg rearing temperature (8.3 ± 1.1 °C during yolk-sac absorption in the current study) rather than diet alone that had a greater influence on the initial development of jaw pathology. In this respect, dietary P deficiency during freshwater grow-out may further exacerbate already pre-existing jaw malformation in triploids. Furthermore, Amoroso et al. (2016b) also demonstrated cartilage impairment and associated gene (*col2a1*) downregulation in LJD affected triploids, and that prevalence of LJD was greater in triploids experiencing higher water temperatures during ongrowing (Amoroso et al., 2016a). Thus, given bone that mineral content of the jaw was not different between diets in this study, this supports the hypothesis that dietary minerals may be sufficient for jaw formation, but that cartilage impairment of the lower jaw formation, particularly in response to rearing temperature, is the primary cause of jaw malformation through incorrect architectural arrangement.

Spinal deformities were examined by a combination of whole body staining in fry and radiological assessment at parr and smolt stages. Diet in triploids affected vertebral ossification in 3 week old fry in the cranial and caudal trunk (R1-R2) based on visual assessment of calcium stained specimens. There appeared to be a greater degree of ossification in MP and HP diets (7.7–9.7 g available P kg^−1^) at 3 weeks than LP fed triploids, although effects of body size cannot be fully excluded (triploid LP specimens were smaller than MP & HP siblings at 3 weeks). However, although LP specimens represented were smaller than MP & HP specimens, these were all collected after the same developmental duration (i.e. same degree days post-feeding). In this respect, LP fed triploids also grew slower than MP and HP in the first few weeks of development and could support the hypothesis that triploids have a higher dietary P requirement for both growth & skeletal development in early life stages. Significantly smaller vertebral areas in the tail region (R3) and tail fin (R4) were in triploids were also evident at 8 weeks when fed 4.9 g available P kg^−1^ (LP) compared to their dietary siblings fed 7.7 or 9.7 g available P kg^−1^ (MP & HP diets) Critical formation of the foundations of skeletal development occurs at this life stage along the cranio-caudal axis through progressive formation of the chordacentra, cancellous bone and trabeculae (Nordvik et al., 2005) indicating a particular sensitivity early developmental period to P deficiency within these regions of the spinal column.

Subsequent radiological examination revealed that the number of deformed vertebrae per deformed individual, although greater in triploids than in diploids at smolt, decreased with increasing dietary P inclusion in both ploidy. Given the higher SGR in triploids compared to diploids in this study in the early life stage, and freshwater as a whole, these findings support previous research linking development of vertebral malformations of triploids with periods of accelerated growth and potential mineral deficiency (Hansen et al., 2010; Smedley et al., 2016). Hansen et al. (2010) also stated that Atlantic salmon presenting <6 radiologically deformed vertebra (dV) are unlikely to be affected significantly in terms of performance, and as such, the population of the current study group were reclassified into two groupings (0–5 dV and ≥6 dV) to examine prevalence of deformity. Triploids fed the LP diet (4.9 g available P kg^−1^) had a greater proportion of the population presenting ≥6 dV than all other treatments at parr (~57%) and smolt (~43%). No further reduction was evident in triploids between MP (7.7 g available P kg^−1^) and HP (9.7 g available P kg^−1^) diets suggesting dietary requirements of P for spinal health may have been met by the medium P level. In diploids at smolt, a comparable proportion of the population with ≥6 dV was evident between 4.9 and 7.7 g available P kg^−1^, reinforcing the idea that diploids have a lower P requirement than triploids for spinal development. By contrast, Fjelldal et al. (2015) did not observe a reduction of the population presenting radiological dV at smolt in triploids when fed between 4.0 and 6.0 g available P kg^−1^, with a significant reduction only evident in triploids fed 12.0 g available P kg^−1^, although dV classification was not presented. By comparison, diploids showed a significant reduction in dV between 4.0 and 6.0 g available P kg^−1^. Collectively, results of the current study would suggest that a comparable effect on skeletal health in triploids can be obtained through using moderate rather than high increases (+2.8 vs. +4.8 g kg^−1^ available P) in dietary P compared to conventional diploid diets (4.9 g kg^−1^ available P). Thus the proposed inclusion level appears to lay between 7.7 and 9.7 g available P kg^−1^ which is inside the range proposed earlier by Fjelldal et al. (2015) of 6.0–12.0 g available P kg^−1^.

Deformities were largely localised in vertebrae numbers 28–30, the cranio-caudal axis for mineralisation along the vertebral column (Grotmol et al., 2003), and 54–57 (region 4) where strong variations in vertebral parameters are typically observed (Kacem et al., 1998). Furthermore, with regards to pathology types observed, symmetry shifts (particularly Type 19) were the most commonly recorded in R2 and R4 in all treatments, as previously reported by Fjelldal and Hansen (2010). However, radio-dense/opaque (Type 11–13) were equally prevalent in triploid LP fish which may indicate under mineralisation and an attempt to strengthen vertebrae or excess cartilage density compared to the neighbouring vertebrae (Helland et al., 2006). Similarly, widely-spaced and undersized vertebral bodies (Type 10) were evident in triploid LP at the parr stage only, which again indicates under-mineralisation of the vertebral body at the early life stage (Witten et al., 2009). Localisation of vertebral malformations are also ontogenetic (Fjelldal et al., 2012b) and the higher prevalence in the central region has been reported in other freshwater studies (Fjelldal and Hansen, 2010; Fraser et al., 2014c; Fjelldal et al., 2015). Parr, irrespective of ploidy, fed a low dietary P inclusion also showed a shorter vertebral L:H ratio compared to MP and HP treatments but not at smolt (with exception of shorter vertebrae in region 3) and a concomitant higher level of malformations were observed in triploids fed low P throughout development. Changes in L:H ratio, while not expressing pathology, may suggest morphological changes in vertebral body construct are occurring (Fraser et al., 2014c). Triploids fed a lower P inclusion had a notably higher number of deformed vertebrae throughout the trunk (R2–R3) particularly at the parr stage, suggesting these malformations may have stemmed from insufficient outward mineralisation in early ontogeny. Malformations in region 3 are commonly observed in SW ongrowing stages and in diploids, are associated with mechanical strain as a result of lateral muscular activity (Witten et al., 2005), low P levels in combination with accelerated growth in the caudal region at smoltification (Fjelldal et al., 2009) and vaccination induced inflammation, which is not prevented by increasing dietary P in post-smolts (Gil Martens et al., 2012). In this study, poorer bone development and mineralisation in triploids fed LP may have been induced through a combination of vaccine-induced inflammation (vaccination performed 8 weeks prior to x-ray assessment), and accelerated early growth under restricted P availability.

Lowered mineral content is associated with reduced structural integrity and abnormal bone development (Fjelldal et al., 2009). Significantly higher whole body P concentration in triploids fed the HP diet than all other treatments suggest a higher accumulation of the bone mineral hydroxyapatite and Ca_3_(PO_4_)_2_ and improved structural integrity, which was reflected in x-ray pathology and whole mount staining results. Higher Zn concentrations in triploid smolts fed the HP inclusion may be attributed to increased activity of metalloenzymes such as alkaline phosphatase, important for formation of osteoid and bone mineralisation (Lall, 2003). V has shown anti-mineralogenic properties in fish bone cells (Tiago et al., 2008), and was found in higher concentration in triploid smolts fed high P, along with increased P concentrations, indicating potential suppression of skeletal mineralisation due to surplus P availability. Lack of differences of P and V in triploids fed HP at the parr stage compared to the other treatments would suggest less active suppression relative to smolt stages and a higher requirement for P in the earlier life stages.

Studies inducing poor mineralisation of vertebrae have indicated linear growth failure resulting in vertebral malformations (Fjelldal et al., 2009) that may occur through insufficient time to mineralise osteoid and production of ectopic cartilage in the intervertebral spaces (Witten et al., 2005). In addition, Fraser et al. (2014a) showed reduced deformity prevalence in triploids in under-yearling S0+ smolts compared to ambient S1 smolts, hypothesising the effect to be due to reduced triploid growth under elevated S0+ thermal regimes. In this respect, the higher deformity rate in the current study (S1+) than that of Fjelldal et al. (2015; S0+) may relate to the difference in thermal regime, and the higher SGRs reported in parr and smolt stages under cooler water temperatures in the current experiment than the former trial. Collectively, the staining, x-ray and environmental temperature results of this study support that triploid require higher dietary P in the very early life stages to prevent linear growth failure and associated vertebral malformation development. However, this may not always be mutually true. A recent study by Witten et al. (2016) fed salmon a P deficient diet for 10 weeks, observing reduced growth but vertebral bodies had a regular size and internal structures, suggesting that deficit in mineralisation may not be the only cause of the alterations and malformation of the vertebral bone structure.

Expression of PO_4_^3−^ homeostasis gene *fgf23* in vertebrae of parr fed LP in both ploidy was significantly lower than HP diets suggesting requirement for mineralisation and minimised excretion of renal PO_4_^3−^. In an earlier study, Fjelldal et al. (2015) also found significantly lower gene expression levels in both diploid and triploid when fed low dietary P inclusion levels albeit at smolt. Conversely, in our study, no differences in *fgf23* expression were observed between ploidy or dietary P at smolt, however, levels of expression were significantly higher than at parr stages. Similarly, Amoroso et al. (2016b) also found no change in *fgf23* between normal jaw and LJD affected triploid smolts. Collectively, this may suggest that early life stages (i.e. parr or fry) are more sensitive to dietary P deficiency if regulated by actions of *fgf23*. Higher levels of available PO_4_^3−^ are known to upregulate *opn* which is associated with activation of ALP and extracellular matrix mineralisation (Beck et al., 2000). Reduced expression of osteogenic factors *opn* and *alp* and skeletal growth factor *igf1r* in triploid parr support lower osteogenic potential in early life stages relative to diploids. A similar response in relation to temperature manipulation and spinal health has previously been reported in diploids (Wargelius et al., 2005). In addition, upregulation of *fgf23* in parr of both ploidy fed the HP diet could promote excretion of renal PO_4_^3−^ and lower circulating levels. However, again lower osteogenic potential in triploids relative to diploids (as reflected in reduced *opn, alp and igf1r* expression) combined with inhibitory factors of mineralisation in the presence of higher dietary P indicate triploids may be subject to lower P availability for skeletal mineralisation. This is supported by other studies that have shown reduced P levels in plasma of triploids fed lower levels of dietary P than their diploid counterparts (Burke et al., 2010). Low availability may be caused by demand in other systemic processes as a consequence of increased growth rate together with reduced digestible efficiency as a result of known altered gut morphology relative to diploids (Peruzzi et al., 2015) and differences in standard metabolic rate and energy utilisation in triploids (O'Donnell et al., 2017).

## 5 Conclusions

Current results clearly show that triploid Atlantic salmon have a higher dietary P requirement than diploids from first-feeding to parr to attain maximum survival, optimal growth and minimise skeletal malformation. Comparable positive effects on skeletal health could be achieved using moderate rather than high increases (+2.8 vs. +4.8 g kg^−1^ available P) in dietary P compared to conventional diploid diets (4.9 g kg^−1^ available P). However, although results of the current study would suggest that dietary P could be reduced from parr stages onwards to promote optimal growth in triploids, it remains to be verified whether the same beneficial long-term effect on skeletal health could be maintained if the duration of increased phosphorus supplementation was reduced. Reducing dietary P inclusion for triploids could present environmental and industrial benefits, including reduced raw feed material costs and lowered risk of freshwater eutrophication from P discharge (Conley et al., 2009; Folke et al., 1994).

The following are the supplementary data related to this article.

Supplementary file A Mean percentage (%) prevalence (±SEM) of deformed vertebrae (n = 2, 50 fish/tank at parr; 35/tank at smolt) in parr (A, B) and smolt (C, D) for diploid (A, C) and triploid (B, D) fish fed low to high P inclusion (LP, MP and HP). Regions 1 (v1–8), 2 (v9–30), 3 (31–49), and 4 (50–60). Supplementary file A

Supplementary file B Vertebral length: dorso-ventral diameters ratios (mean ± SEM, n = 2, 5 fish/tank in both parr and smolt) of vertebrae taken from x-rays at parr (A, B) and smolt (C, D) for diploid (A, C) and triploid (B, D) fish fed low to high P inclusion (LP, MP and HP). Regions 1 (v1–8), 2 (v9–30), 3 (v31–49), and 4 (v50–58/59/60). Supplementary file B

Supplementary file C Nucleotide alignment of predicted fgf23 like sequence XM_014153467.1 and sequenced fgf23 product with protein translation and FGF site, receptor interaction site and heparin binding regions. Supplementary file C
