# Supplementary material for: Impact of dietary phosphorous in diploid and triploid Atlantic salmon (Salmo salar L.) with reference to early skeletal development in freshwater
Source: Aquaculture. 2018 Mar 1;490:329–43. doi: 10.1016/j.aquaculture.2018.02.049 (PMC5905282; doi:10.1016/j.aquaculture.2018.02.049)
Supplement: Supplementary file A — Mean percentage (%) prevalence (±SEM) of deformed vertebrae (n = 2, 50 fish/tank at parr; 35/tank at smolt) in parr (A, B) and smolt (C, D) for diploid (A, C) and triploid (B, D) fish fed low to high P inclusion (LP, MP and HP). Regions 1 (v1–8), 2 (v9–30), 3 (31–49), and 4 (50–60). [file mmc1.docx]

**
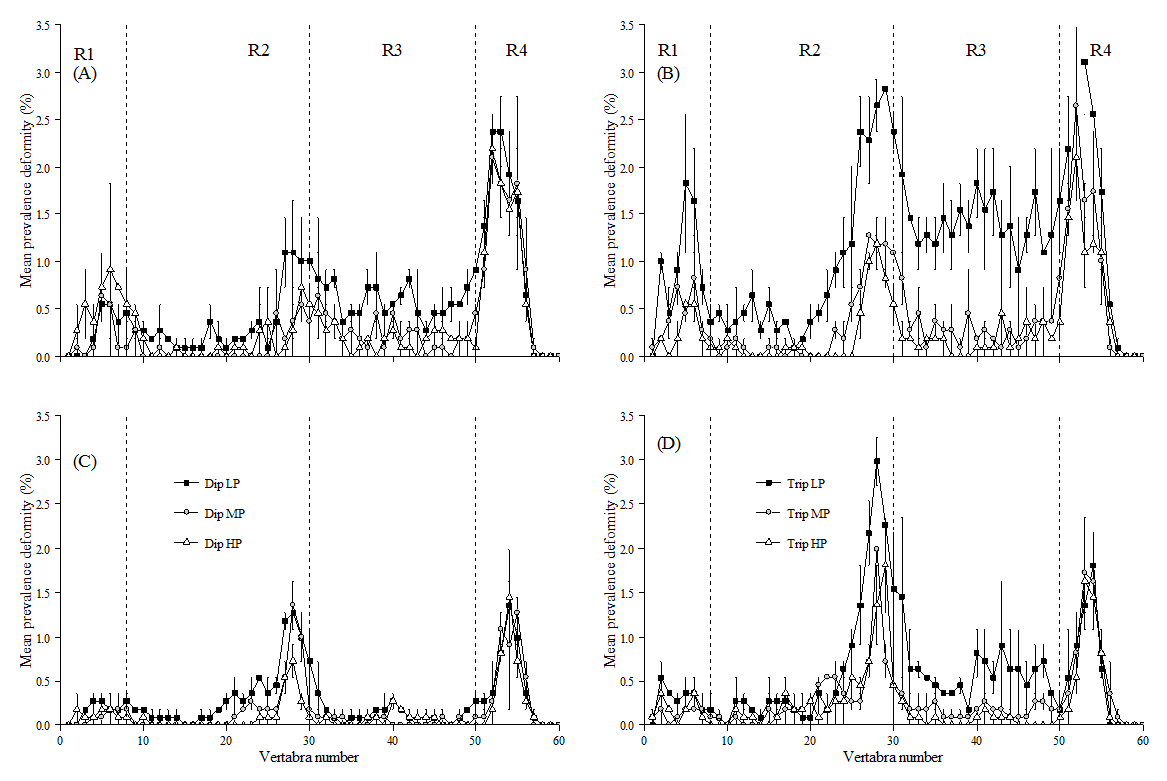
****
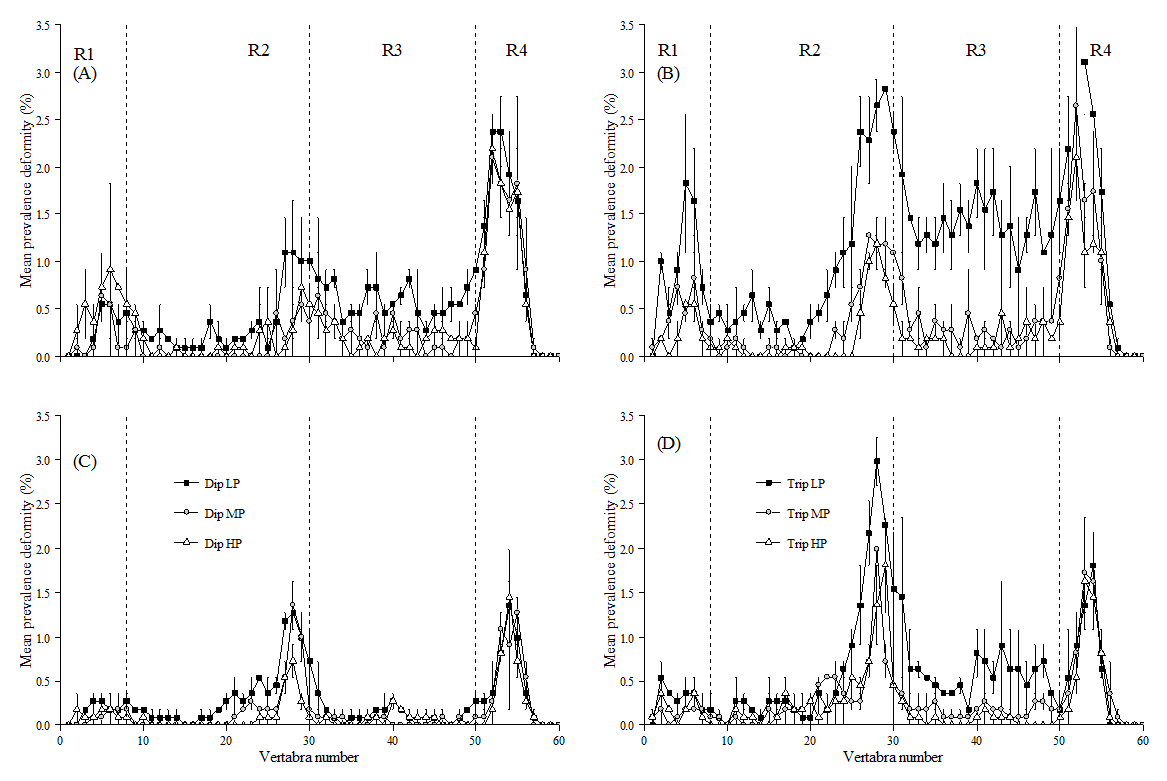
**

**Diploid - Smolt**

**Triploid - Smolt**

**Triploid - Parr**

**Diploid - Parr**

**Supplementary File A.** Mean percentage (%) prevalence (± SEM) of deformed vertebrae (n=2, 50 fish / tank at parr; 35 / tank at smolt) in parr (A, B) and smolt (C, D) for diploid (A, C) and triploid (B, D) fish fed low to high P inclusion (LP, MP and HP). Regions 1(v1-8), 2(v9-30), 3(31-49), and 4(50-60).
