# Supplementary material for: Impact of dietary phosphorous in diploid and triploid Atlantic salmon (Salmo salar L.) with reference to early skeletal development in freshwater
Source: Aquaculture. 2018 Mar 1;490:329–43. doi: 10.1016/j.aquaculture.2018.02.049 (PMC5905282; doi:10.1016/j.aquaculture.2018.02.049)
Supplement: Supplementary file B — Vertebral length: dorso-ventral diameters ratios (mean ± SEM, n = 2, 5 fish/tank in both parr and smolt) of vertebrae taken from x-rays at parr (A, B) and smolt (C, D) for diploid (A, C) and triploid (B, D) fish fed low to high P inclusion (LP, MP and HP). Regions 1 (v1–8), 2 (v9–30), 3 (v31–49), and 4 (v50–58/59/60). [file mmc2.docx]

**Triploid - Parr**

**Diploid - Parr**

**Triploid - Smolt**

**Diploid - Smolt**

**Supplementary File B.** Vertebral length: dorso-ventral diameters ratios (Mean ± SEM, n=2, 5 fish / tank in both parr and smolt) of vertebrae taken from x-rays at parr (A, B) and smolt (C, D) for diploid (A, C) and triploid (B, D) fish fed low to high P inclusion (LP, MP and HP). Regions 1(v1-8), 2(v9-30), 3(v31-49), and 4(v50-58/59/60).
