# Supplementary material for: Impact of dietary phosphorous in diploid and triploid Atlantic salmon (Salmo salar L.) with reference to early skeletal development in freshwater
Source: Aquaculture. 2018 Mar 1;490:329–43. doi: 10.1016/j.aquaculture.2018.02.049 (PMC5905282; doi:10.1016/j.aquaculture.2018.02.049)

**Supplementary File C.** Nucleotide alignment of predicted fgf23 like sequence XM_014153467.1 and sequenced fgf23 product with protein translation and FGF site, receptor interaction site and heparin binding regions.


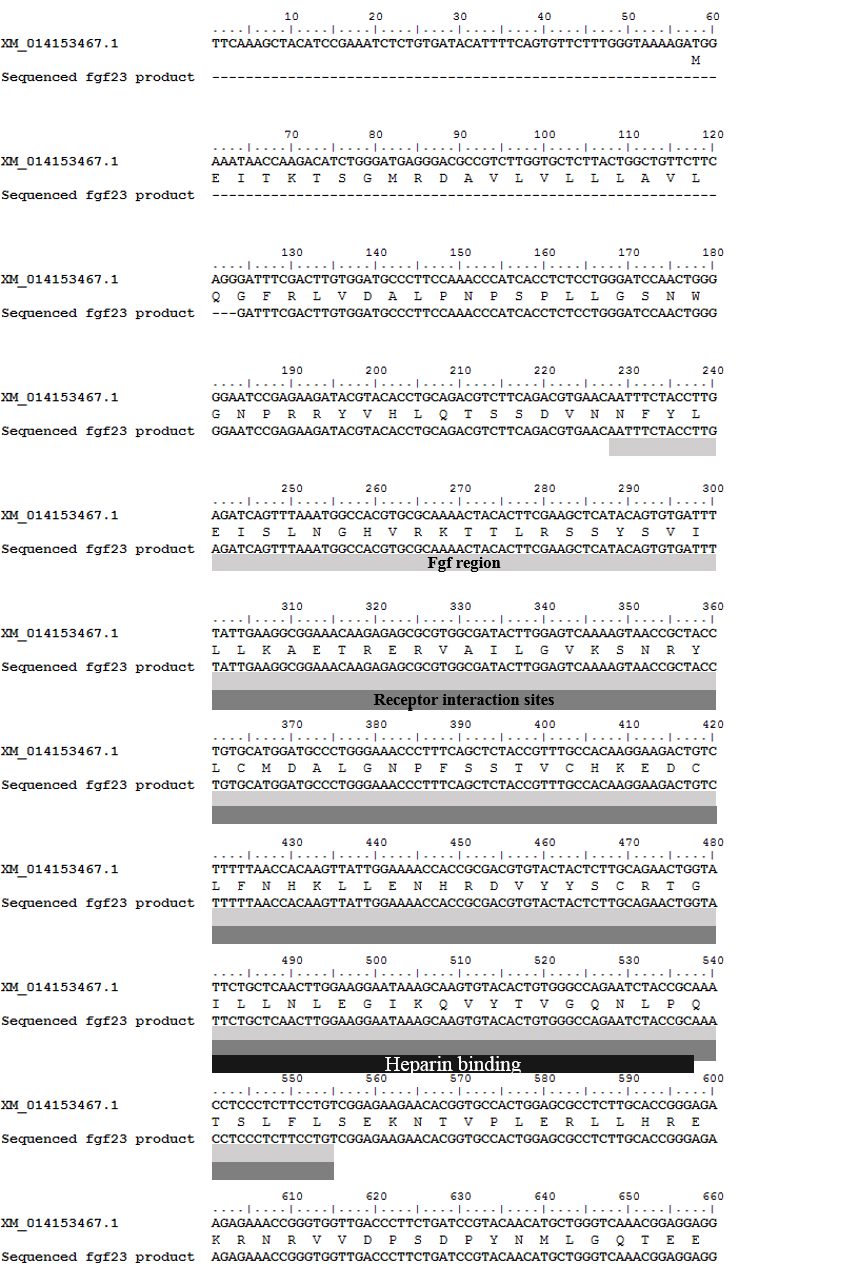

Supplement: Supplementary file C — Nucleotide alignment of predicted fgf23 like sequence XM_014153467.1 and sequenced fgf23 product with protein translation and FGF site, receptor interaction site and heparin binding regions. [file mmc3.docx]
